# Dynamic flood modeling essential to assess the coastal impacts of climate change

**DOI:** 10.1038/s41598-019-40742-z

**Published:** 2019-03-13

**Authors:** Patrick L. Barnard, Li H. Erikson, Amy C. Foxgrover, Juliette A. Finzi Hart, Patrick Limber, Andrea C. O’Neill, Maarten van Ormondt, Sean Vitousek, Nathan Wood, Maya K. Hayden, Jeanne M. Jones

**Affiliations:** 1 United States Geological Survey, Pacific Coastal and Marine Science Center, Santa Cruz, CA 95060 USA; 2 Coastal Carolina University, Department of Marine Science, Conway, SC 29528 USA; 3 Deltares, Delft, The Netherlands; 4 University of Illinois at Chicago, Department of Civil and Materials Engineering, Chicago, IL 60607 USA; 5 United States Geological Survey, Western Geographic Science Center, Portland, OR 97201 USA; 6 Point Blue Conservation Science, Petaluma, CA 94954 USA; 7 United States Geological Survey, Western Geographic Science Center, Menlo Park, CA 94025 USA

## Abstract

Coastal inundation due to sea level rise (SLR) is projected to displace hundreds of millions of people worldwide over the next century, creating significant economic, humanitarian, and national-security challenges. However, the majority of previous efforts to characterize potential coastal impacts of climate change have focused primarily on long-term SLR with a static tide level, and have not comprehensively accounted for dynamic physical drivers such as tidal non-linearity, storms, short-term climate variability, erosion response and consequent flooding responses. Here we present a dynamic modeling approach that estimates climate-driven changes in flood-hazard exposure by integrating the effects of SLR, tides, waves, storms, and coastal change (i.e. beach erosion and cliff retreat). We show that for California, USA, the world’s 5^th^ largest economy, over $150 billion of property equating to more than 6% of the state’s GDP and 600,000 people could be impacted by dynamic flooding by 2100; a three-fold increase in exposed population than if only SLR and a static coastline are considered. The potential for underestimating societal exposure to coastal flooding is greater for smaller SLR scenarios, up to a seven-fold increase in exposed population and economic interests when considering storm conditions in addition to SLR. These results highlight the importance of including climate-change driven dynamic coastal processes and impacts in both short-term hazard mitigation and long-term adaptation planning.

## Introduction

Over 600 million people worldwide live in the coastal zone (<10 m elevation) and migration trends forecast an increase to more than 1 billion by 2050 (ref.^1^). SLR acceleration in recent decades^2^ and median global SLR projections ranging from 0.5 (ref.^3^) to 1.8 m by 2100 (ref.^4^) indicate that growing coastal populations will be increasingly at risk of displacement due to permanent flooding (i.e. inundation), as well as annual flood damages and adaptation costs that could top $1 trillion by the end of the 21^st^ century^5^. Further elevating coastal societal risk is the recent instability of the Antarctic ice sheets^6,7^, indicating plausible SLR up to 3 m by 2100 (refs^4,8,9^).

**Figure 1:**
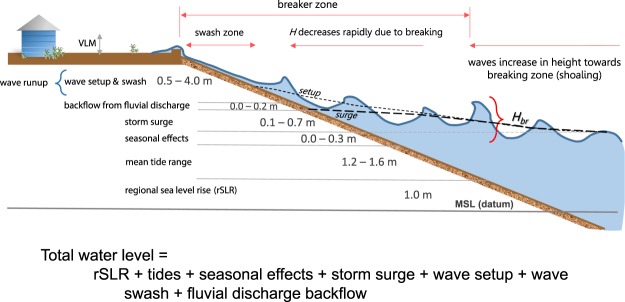
Dynamic water level concept. Example from California for 1 m of sea level rise of the significant water level components that comprise total water levels on a beach during a storm along the coast resulting in potential flooding. The range of values are based on observations and modeling conducted during the development and application of the Coastal Storm Modeling System (CoSMoS) across the region^50,61,89^. (VLM = vertical land motion, H = wave height, H_br_ = breaking wave height).

In addition to long-term SLR, the exposure of the coastal zone population and infrastructure to flooding is amplified during episodic storms, when coastal water levels can increase by several meters or more due to locally-varying combinations of tides^10^, storm surge^11^, waves^12^, river discharge^13^, and seasonal water level fluctuations, as exemplified during El Niño events along the west coast of North America^14^ (Fig. 1). In combination with SLR, these dynamic water level components can disproportionately increase the flood frequency^15^ and volume in the coming decades^16^. To date, most climate-driven, hazard assessments exclude the short- and long-term effects of storms on coastal flooding, beach erosion, and cliff retreat, and instead only account for SLR^17,18^, single components of storm-driven variability^19,20^, or shoreline change due to SLR^21^.

Here we describe a primarily physics-based numerical modeling approach, the Coastal Storm Modeling System (CoSMoS), which was designed to thoroughly assess future coastal flooding exposure by integrating SLR, dynamic water levels, and coastal change. We apply CoSMoS to one of the world’s largest economies and most developed coastal environments worldwide, the urbanized portion of the state of California, USA, which accounts for 95% of the 26 million residents of California coastal counties (2010 U.S. Census Bureau estimate). The model predictions are made available, via interactive web tools that include flood hazard maps and socioeconomic exposure, to support local climate adaptation planning and facilitate large-scale policy action. We show that inclusion of storm-driven dynamic water levels in future coastal flooding assessments (see Fig. 1) results in the additional projected exposure of approximately 200,000 residents and $50 billion in property over the next century compared to SLR alone, as well as significant storm impacts for the lower SLR scenarios. These results illustrate the importance of including dynamic water levels and coastal change in hazard assessments and reinforce the urgency to mitigate and adapt to the expected coastal impacts of climate change.

## Modeling Approach

**Figure 2:**
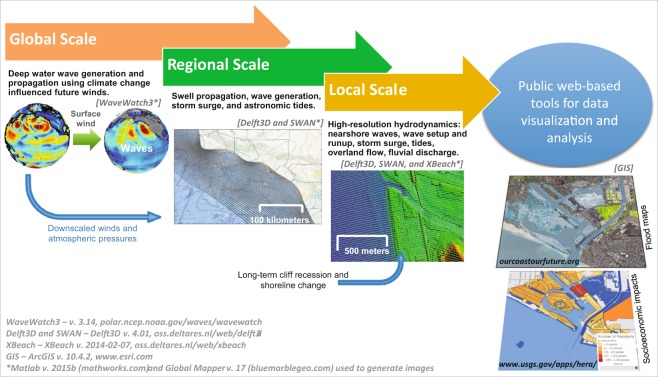
Coastal Storm Modeling System (CoSMoS) workflow. CoSMoS features a series of coupled numerical models that translates the physical forcing derived from Global Climate Models into local coastal flood projections, incorporating sea level rise, tides, seasonal effects, storm surge, fluvial discharge, and waves, as well as short- and long-term coastal change. The hybrid numerical-statistical model is used to develop continuous time-series of total water levels at the shore using a linear superposition of wave runup (maximum excursion that waves reach onshore), storm surge, and sea levels, in contrast to the numerically modeled flood maps which simulate non-linear interactions between changing water depths and waves. For more information on the CoSMoS framework see the Methods section and Supplementary Fig. 1. Figure modified from O’Neill *et al*.^61^. Software citations: WaveWatch3 – v. 3.14, polar.ncep.noaa.gov/waves/wavewatch; Delft3D and SWAN – Delft3D v. 4.01, oss.deltares.nl/web/delft3d with Matlab v. 2015b (mathworks.com) and Global Mapper v. 17 (bluemarblegeo.com) used to generate images.

The overarching concept of CoSMoS is to use a suite of linked oceanographic and geomorphic models (Fig. 2) to assess flood impacts caused by future SLR and storms at management-relevant scales (2 m resolution). CoSMoS utilizes projections of global climate patterns over the 21^st^ century from Global Climate Models (GCMs) developed for the 5^th^ Assessment Report of the Intergovernmental Panel on Climate Change^22^ to determine regional oceanographic conditions. Native resolution GCM projections are dynamically downscaled to the regional and local level and used as boundary conditions for a number of physics-based, numerical ocean models to predict coastal waves, water levels, flooding, and erosion for the range of possible SLR (10 scenarios: 0.00–2.00 m in 0.25 m increments, and 5.00 m, relative to the year 2000) and storm scenarios (4 scenarios: average daily conditions [i.e. including tides and typical wave conditions] and annual, 20-year and 100-year storms) over the 21^st^ Century (Fig. 2, Supplementary Fig. 1, Methods).

The results are provided to the public via two web tools, one focused on physical exposure (Our Coast, Our Future [OCOF]: www.ourcoastourfuture.org)^23^ and the other on socioeconomic impacts (Hazard Exposure Reporting and Analytics [HERA]: https://www.usgs.gov/apps/hera/)^24,25^. Translating the flooding extents into community exposure expresses the consequences of unmitigated coastal hazards in terms of population and property at risk. This is a critical exercise in developing effective return-on-investment strategies to improve coastal infrastructure via beach nourishments, construction of coastal protection structures, improving drainage, and/or managed retreat. Societal exposure to coastal-flood hazards due to the various storm and SLR scenarios were estimated based on several societal indicators, including developed land, resident and employee populations, parcel values, and roads. A detailed technical description of dynamic flood modeling and geospatial exposure analyses are available in Methods.

## Results

### Physical Exposure

**Figure 3:**
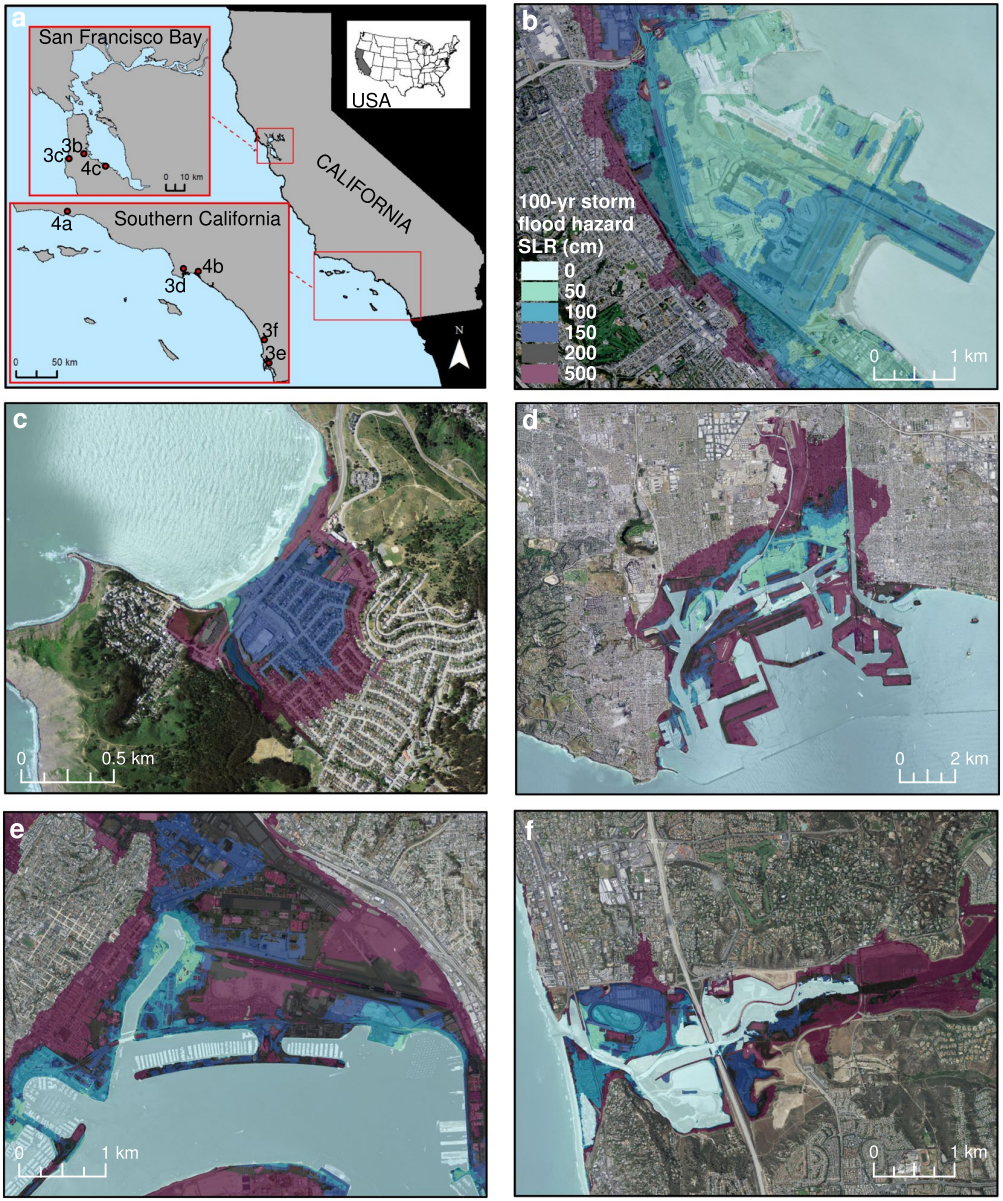
Study area and coastal flooding examples due to an extreme storm. (**a**) Study area for CoSMoS with insets. Examples of modeled flood extents for the 100-year coastal storm in combination with 0, 0.50, 1.00, 1.50, 2.00 and 5.00 m of SLR: (**b**) San Francisco International Airport, (**c**) City of Pacifica, (**d**) Port of Los Angeles and Port of Long Beach, (**e**) Port of San Diego and San Diego International Airport, and (**f**) City of Del Mar. (Figure generated using ArcGIS v. 10.4.2, www.esri.com. Local basemaps from http://services.arcgisonline.com/arcgis/services, World_Terrain_Base and ESRI_Imagery_World_2D, accessed 2 Oct 2018).

Active tectonics have produced a high-relief coastline dominated by coastal bluffs in many locations across California, providing a buffer to SLR flooding not common on passive margin settings like the U.S. East Coast. However, millions of California residents live in or immediately adjacent to low-lying coastal areas and urbanized estuaries, within several meters of present-day sea level. Along the entire study area for the open coast of California, predicted 100-year storm-driven total water levels (see Fig. 1) under present-day conditions average 4.0 m ± 2.8 m (2 standard deviation range, 95%) above MSL (maximum value = 12.6 m). Within the largest estuary, San Francisco Bay, in which waves are much smaller than the open coast, the 100-year water levels average 1.8 m ± 0.8 m (maximum 3.4 m) above MSL. These vulnerable coastal settings often contain important infrastructure, such as airports and ports, which are shown here to be vulnerable to future SLR and extreme storm conditions (Fig. 3).

**Figure 4:**
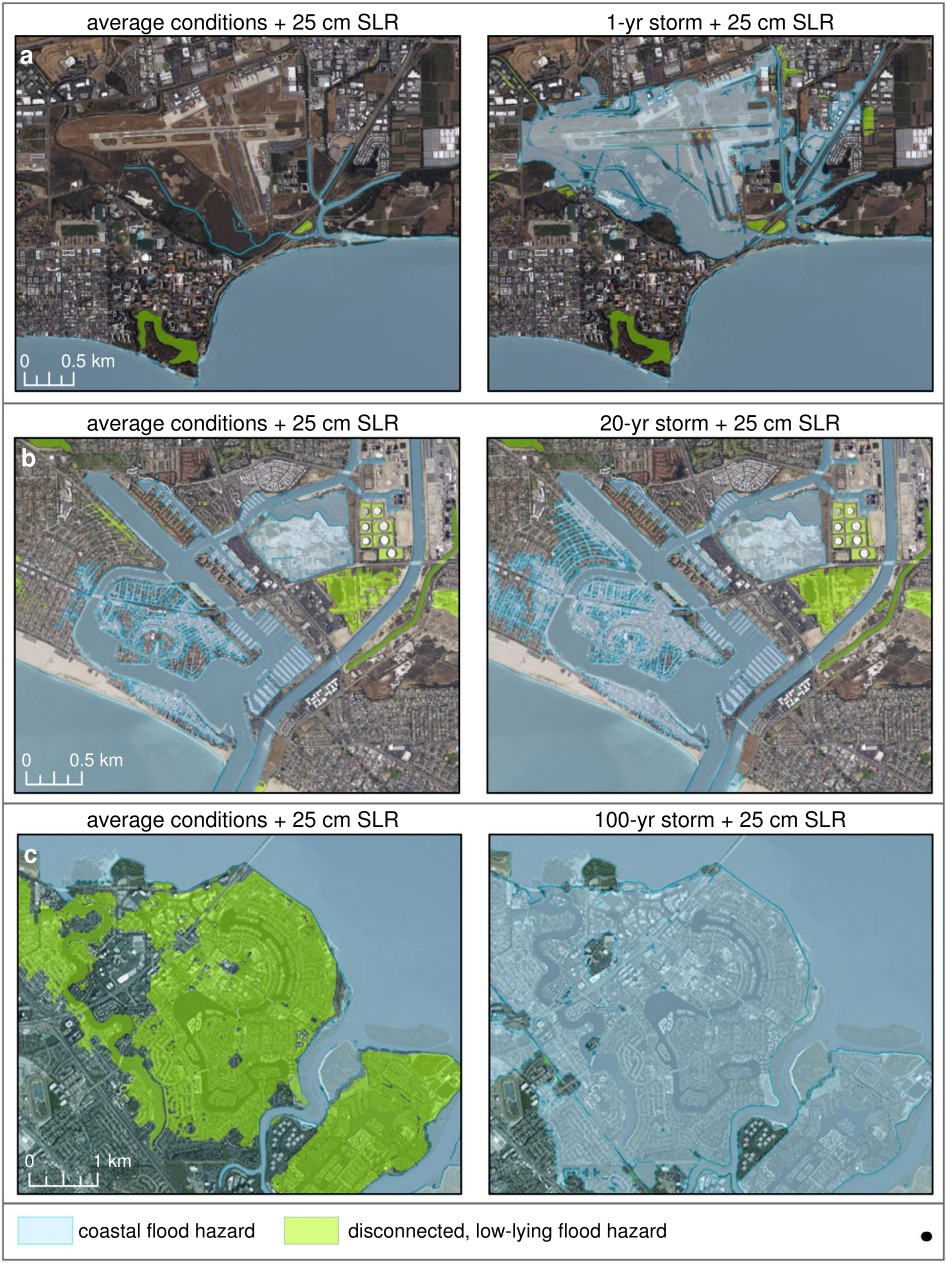
Examples of coastal flooding with 0.25 m of sea level rise and storms. These examples illustrate that there are locations with significant flood risks for small amounts of sea level rise when storms are considered. The left hand series of panels depicts projected coastal flood extent during average conditions (i.e. daily/background conditions with spring tide), and the right side select storm scenarios: (**a**) Santa Barbara Municipal Airport, (**b**) Alamitos Bay, Long Beach, and (**c**) Foster City. See Fig. 3 for locations. “Disconnected, low-lying flood hazard” designates areas that are below the flood elevation surface but are not hydraulically connected to the flooding due to a flow impediment (e.g. levee), and therefore subject to flooding should the flood barrier fail. See Supplementary Fig. 2 to see the uncertainty range for each of the scenarios. (Figure generated using ArcGIS v. 10.4.2, www.esri.com. Local basemaps from http://services.arcgisonline.com/arcgis/services, World_Terrain_Base and ESRI_Imagery_World_2D, accessed 2 Oct 2018).

Many low-lying, exposed coastal areas are currently protected by levees or other defenses designed to withstand historical storm conditions. However, these defenses provide marginal protection against SLR and even less protection against the combined effects of storms and SLR, as most were designed without allowances for future conditions^26^. Our results demonstrate that many sensitive areas may be overwhelmed during storm conditions combined with small amounts of SLR expected within just a few decades (Fig. 4, Supplementary Fig. 2).

**Figure 5:**
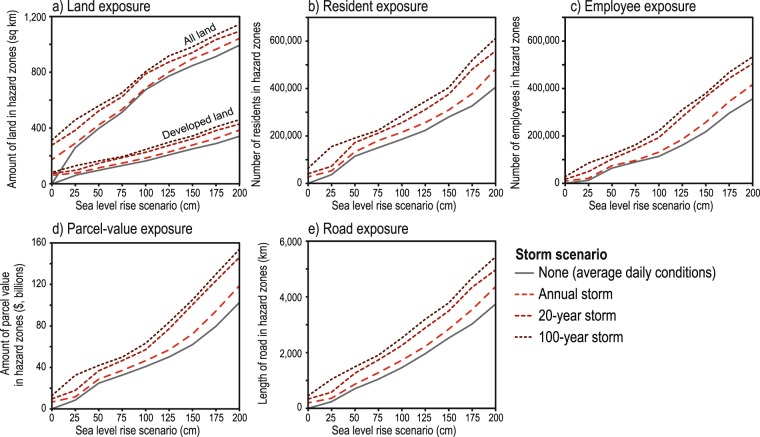
Absolute changes in exposure to coastal-flooding hazards. Absolute changes in flooding exposure based on variations in sea level rise and storm scenarios for: (**a**) land, (**b**) residents, (**c**) employees, (**d**) parcel value, and (**e**) roads for the California study area. All values are in 2010 U.S. dollars.

Across the study area, 1.00–2.00 m of SLR is projected to permanently inundate 670–990 km^2^ of land (flood potential/uncertainty range = 430–1,220 km^2^), and an additional 15–19% of land would be flooded during a 100-year storm (Fig. 5). However, for the 0.25 m and 0.50 m SLR scenarios, 48% and 32% more land, respectively, would be projected to flood during the 20-year storm compared to that inundated solely by SLR. During the 100-year storm, 77% and 41% more land would be flooded compared to SLR alone. Based on current SLR trajectories and the latest regional SLR projections for California, intermediate scenarios suggest 0.25 m and 0.50 m of SLR may be reached by the 2040 s and 2060 s, respectively^9^.

Projections of long-term coastal change driven by a 21^st^ century total water level time series including each of the SLR scenarios are integrated into the coastal flooding scenarios across the most populated part of the state, southern California (17 million coastal residents, 71% of total study area) (Supplementary Data, Supplementary Fig. 3). By 2100, 1.00–2.00 m of SLR would result in an average projected beach loss of 26–41 m across this portion of the study area (95% confidence range = −11 to 93 m), completely eroding up to 67% of the beaches^27^. Bluff retreat projections by 2100 are 19–30 m for SLR ranging from 1.00–2.00 m (95% confidence range = 13 to 38 m), with an increase in retreat rates of 180% for the 2.00 m SLR scenario as compared to the historical rates in southern California^28^. Lower SLR scenarios result in less but not insignificant erosion: for example, 0.50 m of SLR results in 14 m of beach loss and 11 m of cliff retreat. An additional 17–36 m of beach erosion is predicted during the storm simulations (Supplementary Fig. 3). These model projections assume existing shoreline infrastructure remains in place.

### Socioeconomic Exposure

**Figure 6:**
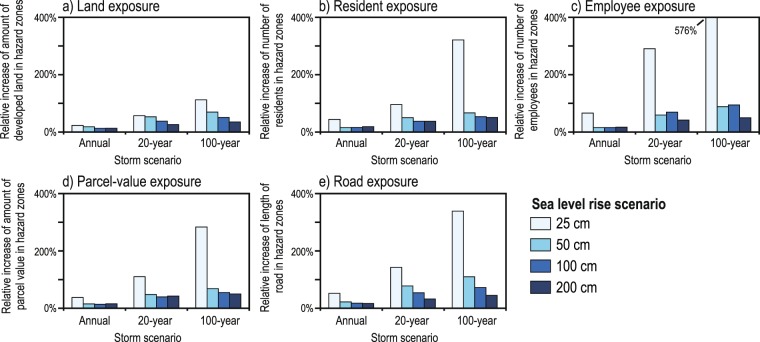
Relative changes in exposure to coastal-flooding hazards. Relative changes in flooding exposure based on variations in sea level rise and storm scenarios for: (**a**) land, (**b**) residents, (**c**) employees, (**d**) parcel value, and (**e**) roads for the California study area. Percentages note relative increases in exposure due to the inclusion of storm conditions compared to hazard exposure based solely on select sea level rise scenarios (i.e. 0.25 m, 0.50 m, 1.00 m, and 2.00 m). These estimates are based on present-day socioeconomic and land use conditions, and do not account for future economic growth, coastal development patterns, climate change mitigation measures, etc.

Translated to socioeconomic impacts, 0.25–2.00 m of SLR alone (no storm) equates to the flooding exposure of between 37,000–406,000 residents (uncertainty range = 23,000 to 729,000 residents) and 13,000–357,000 employees (uncertainty range = 7,000 to 593,000 employees) (Fig. 5, Supplementary Data). However, with the addition of a 100-year storm to 0.25–2.00 m of SLR, these values increase to 155,000–612,000 residents (uncertainty range = 95,000 to 1,017,000 residents) and 86,000–534,000 employees (uncertainty range = 43,000 to 798,000 employees). For 0.25 m of SLR, the 100 year-storm compared to the no-storm scenario increases the residents and employees at risk by 322% and 576%, respectively, and 51% and 50% for the 2.00 m SLR scenario. While the percentages are smaller at the higher rates of SLR, the absolute number of population affected and economic impacts is far greater than for the lower SLR rates (Fig. 6). The relative increases in population exposure when including dynamic water level components from the annual to 100-year storms are 16–67% for 0.50 m SLR, 16–54% for 1.00 m SLR, and 19–51% for 2.00 m SLR.

The value of property in flood hazard zones due solely to SLR ranges from $8 billion for 0.25 m of SLR to $103 billion for 2.00 m of SLR (uncertainty range = $4 billion to $166 billion), but increases from $32–154 billion when considering the 100-year storm (uncertainty range = $18 billion to $210 billion). The consideration of storm conditions in the dynamic flood projections results in an approximately 4-fold increase (283%) in property exposure for the 0.25 m SLR and 100-year storm scenario. As was the case with other socioeconomic factors, these relative increases are substantially lower for higher SLR scenarios: property value exposure for 2.00 m of SLR and a 100-year storm is only 50% higher than a SLR-only scenario, though the net value of property is much higher with the higher rates of SLR (e.g. +$51 billion for 2.00 m SLR vs +$24 billion for 0.25 m SLR). Similar trends related to changes in hazard exposure due to the inclusion of storm scenarios were also observed for roads and developed land (Figs. 5,6). Here in the results we mainly present the impacts associated with median flood projections, and only the full uncertainty/flood potential range in select instances, but the range for each of the socioeconomic metrics is provided in the Supplementary Data. This analysis takes into account the range in potential flood exposure related to the uncertainty of the underlying elevation data, model accuracy for prediction of total water levels, and vertical land motion (see Methods for more information).

## Discussion

For the vast majority of the urbanized coast of California, the inclusion of storms in coastal flooding projections – in combination with the range of SLR expected by 2100 (i.e. 0.50 to 2.00 m) – increases population and property exposure from 16% for the annual storm to 57% for the 100-year storm compared to the no-storm scenarios (i.e. average daily conditions, including tides, waves and long-term coastal change) (Fig. 6). More than 600,000 people and $150 billion (2010 dollars) are at risk for the 2.00 m SLR + 100-year storm scenario. When factoring in coastal population trends^18^ this extreme scenario could equate to over 3 million residents at risk across the state by 2100. Excluding speculation about future population trends, these projected flood impacts represent 1.6% of the current California population and 6.3% of the state’s GDP, despite only directly affecting 0.3% of the state’s land area. This reflects the disproportionate density of the coastal population (5 times higher) and concentration of coastal property value (20 times higher). However, this example only estimates exposure from a single extreme, 100-year storm: under the same SLR scenario of 2.00 m, the recurring annual storm, which is more relevant to emergency response planning, is estimated to expose 483,000 residents (based on 2010 census data) and $119 billion (2010 dollars) in property by 2100. The economic impacts of projected future coastal flooding in California are of the same order of magnitude as estimates (all in 2010 dollars) from two of the costliest recent natural disasters in the world, the Tōhoku Earthquake and Tsunami ($325 billion)^29^ and Hurricane Katrina ($127 billion)^30^, and an order of magnitude higher than the most costly natural disasters in California history, the 1989 Loma Prieta Earthquake ($10 billion)^31^ and the 2017 Wildfire Season ($18 billion)^30^. A future hypothetical but scientifically-plausible megastorm, the ARkStorm, which was modeled to approximate the historic flooding in 1861-62, would cause catastrophic inland flooding across California and property damage of over $300 billion^32^. This comparison suggests to policy makers that future coastal flooding due to storms and sea level rise must be considered an economic threat on par with the state’s and world’s most costly historical natural disasters.

Furthermore, the alarming scale of these impacts does not account for the ripple effects such extreme events have across economic sectors such as those related to closures of ports, disruption of transport of goods and services, business closures, and impairment of utilities both today and into the future^33,34^. As demonstrated by the impact of severe storms throughout the Gulf Coast and Caribbean in 2017, these disruptions impact critical lifeline services (e.g. water, power, sewage, public health, transportation, fuel and communication) essential for public safety and community stability. Indeed, the US Department of Defense (DoD) identified climate change, and its ensuing impacts, as a potential “threat multiplier” that puts geopolitical stability at risk globally^35^.

The cost of adaptation can be high, particularly for the ports, which are a critical part of the economy; for instance, the Ports of Los Angeles and Long Beach alone handle $478 billion in cargo annually (3% of national GDP) and support 2.8 million jobs across the United States (2 out of every 100 jobs, including 1 in 9 in the greater Los Angeles area)^36,37^. The cost to elevate and retrofit the major commercial ports of California (i.e. San Diego, Los Angeles/Long Beach, and San Francisco) to adapt to 2.00 m of SLR is $9–12 billion^38^. Equally, local impacts along the California coast can have cascading economic impacts both nationally and globally^33,34^. Beyond the potential physical impacts to the port terminals and harbors that could impact the U.S.’s ability to accept imports, coastal flooding and erosion will impact rail lines and roads exiting the ports, disrupting the movement of goods out of ports to other regions throughout the U.S.^39^. Hence, targeted adaptation efforts will be critical to ensuring economic continuity in a changing climate.

Along the vulnerable shoreline of San Francisco Bay, which accounts for two-thirds of the flooding impacts projected for California, building defenses to withstand 2.00 m of SLR and a 100-year storm could cost up to $450 billion, almost twice the cost of defending against SLR alone^40^. There is also a non-linear increase in costs to defend against the higher SLR projections, as costs are approximately 3-4 times higher for the 2.00 m SLR scenario as compared to the 1.00 m scenario. This highlights the need for the continual effort of scientists to improve estimates of 21^st^ century SLR curves.

Previous efforts to characterize coastal impacts from climate change often focus on high SLR assumptions on the order of 1-2 m^18^ that are most likely expected around the end of the century. In our study area, under the most extreme SLR projection, 1 m of SLR could arrive as soon as 2060 (ref.^9^). However, there is greater confidence that 0.25 m of SLR will be reached by ~2040. Our work here shows that an extreme storm (i.e. 100-year storm) in combination with even this relatively low amount of SLR would cause substantial flooding that would directly affect over 150,000 residents and $30 billion in property in California, a 4-fold increase over the impacts projected by only SLR.

Notably, for any of the socioeconomic factors, the relative increase in storm-related flooding exposure under the lower SLR scenarios is greater than at the higher SLR scenarios, as storm driven water levels represent a larger percentage of the total water level in the former case. For instance, there is a nearly 7-fold increase (576%) in the number of employees exposed to flooding with 0.25 m of SLR at the 100-year storm versus no storm; this is in comparison to only a 50% increase at 2.00 m of SLR. Similarly, with only 0.25 m of SLR projected to occur by ~2040, the number of residents exposed to flooding from an annual storm is expected to double compared to year 2000, and increase five-fold for 0.5 m of SLR. Although the net number of people impacted with 0.25 m of SLR is less than those impacted with 2.00 m of SLR, emergency managers do not currently plan for increases of this magnitude. Local hazard mitigation plans – the main planning documents that outline a municipality’s strategies to reduce risk to natural and man-made hazards – generally only forecast out 25–30 years (with updates every 5 years) and until recently have generally been based on historical and current day exposure. Although there are examples of emergency managers in California that are incorporating projections of the 100-year storm in tandem with SLR^41,42^, many still do not and they therefore underestimate their community’s risk, particularly under low rates of SLR.

Compared to an earlier study for California that only accounted for SLR and tidal flooding on a static coastline^18^, the addition of long-term coastal change (which for this summary was only completed for the southern California study area) modeled here produces twice the population at risk when comparing similar 2100 SLR scenarios with no storm (i.e. 0.90 in the prior publication vs 1.00 m in this study, and 1.80 vs 2.00 m). Including dynamic water levels and storm-driven beach erosion for the 100-year storm with long-term coastal change for both the 1.00 m and 2.00 m SLR scenarios tripled population risk compared to the prior study. This suggests that first-order studies of climate impacts that do not account for dynamic water levels and shifting coastlines may vastly underestimate hazard risks to coastal populations over the next century. Further, the application to California described herein is for a relatively high-relief coast, but the additional exposure due to storms and coastal change could be far greater for low-lying coastal settings that are already highly susceptible to coastal flooding due to SLR, such as the majority of coastal megacities^43^ and island nations^44^. Therefore, to better assess the true risk of climate change to global coastal communities, a dynamic approach should be applied that projects long-term beach and cliff evolution and integrates those changes with a plausible range of SLR and storm scenarios.

The importance of dynamic modeling is further illustrated when considering non-linear feedbacks and non-stationarity of the physical drivers of extreme water levels (e.g., tides, surge, and waves), particularly in shallow estuaries and open coastal settings. For example, in San Francisco Bay over the 20^th^ Century, the Mean Higher High Water tidal datum rose 26 cm, outpacing mean sea level rise by ~16%^45^. In addition, recent hydrodynamic modeling in San Francisco Bay indicates that measureable tidal amplification occurs on the order of ~5 cm if present-day shorelines are maintained and up to ~30 cm if seawalls are built in the future for 1 m of SLR, whereas dampening of up to ~10 cm could occur by allowing flooding into intertidal regions that would serve as energy-absorbing sinks^46,47^. At broader scales, observations at over 150 tide gauges across the Pacific Basin show a significant correlation between SLR and tidal extremes^48^, and therefore the non-stationarity of tides and non-linear feedbacks within tidal basins needs to be considered in the modeling of future extreme water levels.

Along the open coast, a common practice is the linear superposition of extreme water level components to assess coastal hazard risk and establish coastal protection design requirements. However, shallow coastal areas are extremely sensitive to non-linear feedbacks between SLR and waves in particular due to an increase in shelf and nearshore water depth and a correlative reduction in frictional dampening that can also affect tides and surge^49^. Therefore, a dynamic modeling approach that includes morphodynamic response, depth-limited breaking, and wave-current interaction, as described in this manuscript, is essential to capture those non-linear feedbacks and properly assess future coastal hazard risk.

### Study limitations and future work

The CoSMoS modeling system is a comprehensive, physics-based approach for determining coastal flood exposure in dynamic, high-energy open coastal and estuarine environments. While the scientific approach has been heavily vetted^27,28,50–61^, like any model it is imperfect, with key limitations, a few of which are discussed here. First, the wave climate and derivation of future storm conditions is based on a series of four GCMs from the CMIP5 suite of models^22^ and 2 Representative Concentration Pathways (RCP) scenarios (4.5 and 8.5) that project atmospheric conditions to 2100. While we have tested and utilize GCMs that yield the best results compared to observations of wind, pressure and waves for the California coast during the hindcast period, past fidelity does not guarantee future performance. Each of over 40 commonly used GCMs provides but one possible realization of the future climate based on unique internal model physics and an assumed emissions trajectory. Therefore, the accuracy of the wave and storm climate derived from each realization is highly uncertain and difficult to quantify. Further, unlike tropical cyclones which are not resolved, the representation of the El Niño Southern Oscillation (ENSO) in CMIP5 GCMs has advanced but projections of the magnitude and frequency of future end member events, El Niño and La Niña, varies widely across GCMs^62–66^. The precise 21^st^ century behavior of ENSO, which is the dominant control on short-term climate variability and coastal hazards across the Pacific Basin^67^, will play a significant role in the timing and frequency of extreme flooding events when coupled with SLR. In addition to eustatic SLR, the uncertain future evolution of the time-varying spatial distribution of sea level across the Pacific Basin due to factors such as ENSO^68^ and the Pacific Decadal Oscillation^69^ will also contribute to local coastal hazard risk. Future work in leveraging the new CMIP6 suite of models may provide a more accurate representation of 21^st^ century climate variability and storm conditions, and continued advances in computational efficiency and ensemble projections can utilize a larger volume of models and RCP scenarios in developing wave climates and uncertainty estimates. Similarly, atmospheric rivers (ARs)^70^ are poorly resolved in the older generation of GCMs due to their narrow width (~100 km), and while not associated with extreme wave conditions they do account for 15–50% of annual storm surge maxima along the U.S. West Coast^71^. Therefore, the effect of ARs on flooding in estuaries (in particular San Francisco Bay) where storm surge is a larger component of extreme water levels, may be underrepresented. A current limitation of CoSMoS is also its coupling with fluvial discharge, which is currently done via a 1-D, one-way coupling, where discharge rates are determined based on an empirical relationship between atmospheric conditions and discharge data, where it exists^58,61^. A dynamic coupling with a watershed-based model that incorporates fluvial and coastal current interaction, wave and surge penetration, locally-downscaled future precipitation trends from GCMs and time-dependent factors that influence flow rates such as seasonal precipitation and soil conditions would surely improve flooding projections in these locations.

Communities along estuaries are highly vulnerable to present-day and future coastal flooding, with the low-lying San Francisco Bay Area accounting for two-thirds of socioeconomic impacts across California in this study. These communities are protected by hundreds of kilometers of levees, but while they are assumed in our modeling approach to be stable, the engineering integrity of most of these structures is poorly understood. The same follows for coastal protection infrastructure (e.g. revetments, sea walls, berms) across the state in smaller estuaries and on the open coast. There is no accommodation for the potential failure of these structures when stressed by future flooding events, yet some will undoubtedly fail and expose more residents and assets to flooding than estimated here. Future work would benefit from a thorough engineering analysis of the potential for flood protection structure failures. In addition, there are other flood protection structures and flow conduits important to local coastal flooding patterns that are typically beyond the resolution of this modeling approach, such as tide gates, culverts, sewage outflows, and narrow sea walls. Greater computational power and sub-grid resolution modeling in future work will enable hydrodynamic models to resolve more of the important, small-scale topographic features that control flooding.

Finally, a more robust assessment of uncertainty is a major challenge and need for future work to provide stakeholders with the most accurate coastal hazards risk assessments possible. Presently, the flood exposure uncertainty is based on just a few, easily quantifiable parameters: topographic data elevation accuracy, model skill in predicting water levels at tide gauges during hindcasts, and projections of vertical land motion based on models and recent observations (see Methods). However, there are many other sources of uncertainty that directly affect the modeling results, including the future wave climate, ENSO variability, model skill in deep-water wave transformation to the nearshore (especially wave height and direction), beach morphology (especially slope), wave set-up and run-up, long-term coastal change, timing of storm during the tidal cycle, etc. Whereas state-of-the-science tools have been used to simulate these processes in the research described herein, highly accurate representations of future conditions remain a challenge. Uncertainty in the coastal change projections has been determined (see Supplementary Data) but not carried through to the storm scenarios runs due to computation expense. Socioeconomic impacts are based only on the flood uncertainty, but those figures have their own inherent uncertainty based on present-day data limitations. Including estimates of future population trends, land use patterns, and economic conditions would be optimal, but further add to the complexity of the uncertainty analysis. In short, while CoSMoS accounts for the primary physical processes that contribute to future coastal flooding, there are a series of research paths that could be pursued to improve model performance and uncertainty analysis, enabling end-users to make more informed coastal management and climate adaptation decisions.

## Methods

### CoSMoS modeling framework

To address the non-stationarity of the future wave climate, global wind fields from four GCMs, driven by 21^st^ century climate change scenarios derived from the Coupled Model Intercomparison Project Phase 5 (ref.^22^), are fed into the WAVEWATCH III (WWIII)^72^ global wave model (Supplementary Fig. 1). A higher resolution Eastern North Pacific WWIII model is nested within the global WWIII model to produce 21^st^ century wave conditions at the edge of the continental shelf driven by winds from a single GCM (i.e. GFDL-ESM2M) and the RCP 4.5 climate scenario^51^. Regional wave conditions for individual storm events identified *a priori* (see following sections on identifying storm events) are then fed into nested, higher resolution SWAN^73^ wave models that dynamically downscale both swell waves from the WWIII model and wave growth due to winds across the shelf to shore. Coupled to these wave models are a series of nested DELFT3D-FLOW^74^ hydrodynamic models that downscale the astronomic tides, seasonal water-level anomalies, storm surge and local river discharge from downscaled atmospheric pressure and wind fields^56,75^ across the shelf and at the coast. Grids at a resolution of ∼10–20 m simulate overland flows in complex coastal settings, such as along the margins of estuaries, harbors and river mouths. Along the exposed open coast, XBeach^76^ profile models, with a cross-shore resolution of 5 m at the shore, are applied every 100–200 m in the alongshore direction to simulate event-driven shoreline change, wave set-up, and swash (i.e. run-up). In contrast to SWAN, the XBeach model computes wave set-up and swash from both incident and infragravity wave energy, the latter which is a dominant component of storm-driven water levels on dissipative beaches^12^. Open boundary conditions for the XBeach models consist of time-varying Jonswap wave spectra and variations in water level due to tides, storm surge, and sea level anomalies extracted from the SWAN and DELFT3D-FLOW models along the 10 to 15 m depth contour. To include appropriate river discharge that may occur during the coastal storm events, river discharge rates are estimated from the atmospheric pattern in a given storm event and are included as point source discharges in the DELFT3D-FLOW model^58,61^. Predicted flood levels are interpolated onto regularly-spaced grids and subtracted by a 2-m resolution digital elevation model (DEM) to isolate areas that are not hydraulically linked to the ocean but were incorrectly flooded in the coarser-resolution numerical model. The DEMs were developed using nearshore multibeam bathymetry soundings and topographic LiDAR (Light Detection and Ranging) data^77^, providing a seamless elevation surface for the numerical hydrodynamic flood models. The DEMs also provide the initial geomorphic conditions for the long-term coastal change models (described below) that are integrated into the flood projections.

The computational expense of the full CoSMoS modeling system (coupled WWIII-SWAN-Delft3D-XBeach) prevents the generation of a continuous 21^st^ century time-series for the entire region, and therefore a proxy approach was developed to identify storm scenarios that were subsequently simulated in full detail with the CoSMoS system^59^. A total water level time-series with three-hour resolution were first computed at thousands of individual points along the coast every ~100 meters by assuming a linear superposition (simple adding, not accounting for non-linear interactions) of the primary storm-driven water levels at the shore, i.e. storm surge, sea level anomalies, and wave-run-up. Empirical models were used to estimate storm surge, sea level, and wave runup levels^12,59^. Annual, 20-year, and 100-year return period storms were then identified from each of the 100-year long total water level time-series spanning the 21^st^ century by identifying peak events at least 3 days apart and ranking these events. Space and time-varying swell waves (from the WWIII model) and downscaled atmospheric wind and pressure fields associated with each identified storm event were then used as boundary conditions to drive the full CoSMoS model system and simulate individual storms.

Two newly-developed, data-driven models were used to simulate long-term cliff retreat^28^ and sandy beach evolution^27^ at ~5000 cross-shore transects spaced every 100 m along the southern California coast. Coastal cliff retreat is projected using a multi-model ensemble that includes vertically-discretized cross-shore models^78–80^, as well as empirical and statistical models that scale wave forcing and SLR to time-averaged cliff edge retreat rates^81–83^. At each transect, the ensemble gives preference to models that show less sensitivity to variations in model parameters based on the standard deviation during Monte Carlo simulations, and then weights projection uncertainty proportionally with the difference between individual model results (i.e. how well the ensemble reaches a consensus)^28^. The CoSMoS-COAST shoreline change model^27^ combines three process-based models to compute sandy beach change: (1) wave-driven longshore transport^52^, (2) cross-shore transport due to waves^84^, and (3) cross-shore transport due to SLR^85^. Both the cliff and sandy shoreline change models use historical shoreline positions, hindcast nearshore wave conditions (wave height, period, and direction), and an adaptive data assimilation scheme to calibrate a suite of equations and develop relationships between wave forcing parameters and geomorphic change at each model location. To drive both cliff retreat and shoreline model projections, continuous time-series of projected nearshore waves^53^ and water levels^54^ through the 21^st^ century, combined with different sea-level rise rates, are used through the year 2100. This is the same total water level proxy that is used to identify storm events^59^. The two models provide time-varying sandy shoreline (mean high water [MHW] line) and cliff positions (cliff edge) that are subsequently used to evolve cross-shore profiles^55^ extracted from the originating high-resolution DEM; evolved profiles are used in scenarios that incorporate future SLR and storms using full model physics of the CoSMoS flood model described above. A summary of the coastal change results is shown in Supplementary Data and Supplementary Fig. 3.

The suite of model projections includes flood extent, depth, duration, uncertainty, water elevation, wave run-up, maximum wave height, maximum current velocity, and long-term shoreline change and bluff retreat. Uncertainty in the system is represented by a vertical offset value calculated by combining the root-mean-square errors between modeled and measured total water levels (from tide gauges during historical storms), the accuracy of the elevation data used to develop the DEM (±18 cm), and vertical land motion as derived from Interferometric Synthetic Aperture Data^86^, GPS data, and/or statistical and physical wetland^87^ and tectonic models^88^ (variable per scenario). While models compared favorably to regional observation stations (*rmsd* and *bias* <6 cm)^61^ for tested conditions, model uncertainty is represented by a larger value (±50 cm) to address the limited number of tested observations compared to the size and complexity of the region. This total system uncertainty in the CoSMoS framework is used to produce spatially-varying flood potential for each scenario (maximum/minimum flood extent given total uncertainty), providing amplifying information on potential vulnerability. More detailed information on the CoSMoS methods can be found in these references^27,28,50–61^.

### Physical exposure web tool

The model results are freely available for download from a public server^89^; however, this static repository of 100 s of gigabytes of high-resolution data is ineffective for public engagement and community use. To better communicate impacts to the variety of community stakeholders reliant on this project, physical exposure results from the 40 scenarios are served up on a public-facing, interactive web tool, Our Coast, Our Future (OCOF)^23^. The OCOF web tool provides coastal managers and the general public a user-friendly means to visualize how future scenarios of coastal flooding will impact local roads, property, businesses and critical utilities. Users can also export informational tables and reports detailing changes in flood extent by scenario on a scale relevant to local planners. Because CoSMoS does not estimate when a scenario will occur, the OCOF tool provides users an interactive comparison of California state guidance and other best available estimates to consider when levels of SLR are expected to happen.

### Societal exposure to flood hazards

Societal flood exposure was estimated based on the geospatial analysis of CoSMoS hazard zones (Supplementary Fig. 4) and various socioeconomic indicators (Supplementary Data). All data sources and supporting references are fully summarized here^24,25^. In short, residential population is based on counts in 2010 Census block data and employee locations and counts are from the 2012 Infogroup Employer Database. Total assessed parcel values, including improvements and land, were obtained from individual county tax assessor offices. Land cover comes from 30-m resolution data of the 2011 National Land Cover Database. Road data were obtained from the Homeland Security Infrastructure Program. Polygons (e.g. census block, parcel values) that partially overlap hazard zones were taken into account during analysis and final values were adjusted proportionately. Ranges in socioeconomic indicators due to modeling uncertainty are displayed both spatially and graphically in the web application, and summarized in the Supplementary Data. Exposure estimates are based on current socioeconomic data and not future projections^18^ due to the high amount of existing development already in hazard zones along the California coastline and the possibility that future growth patterns may vary from historical trends as water levels rise in coming decades. Realistic projections of future urban growth would require local understanding of risk tolerance and carrying capacity for additional growth in hazard zones, which were considered outside the scope of this analysis.

### Code availability

The models and software tools used to generate the results for this project are available upon request from the corresponding author.

## Supplementary information

Supplementary Figures

Dataset 1

## Data Availability

The model projections used in the production of this manuscript are available at the USGS *Science Base* website (10.5066/F7T151Q4) and also served up and downloadable via the *Our Coast, Our Future* interactive web tool (https://www.ourcoastourfuture.org). The socioeconomic projections are available and downloadable via the interactive *Hazard Exposure Reporting and Analytics (HERA)* web tool (https://www.usgs.gov/apps/hera/). Any additional datasets generated during and/or analyzed during the current study are available from the corresponding author on reasonable request.
