# Supplementary material for: Dynamic flood modeling essential to assess the coastal impacts of climate change
Source: Sci Rep. 2019 Mar 13;9:4309. doi: 10.1038/s41598-019-40742-z (PMC6416275; doi:10.1038/s41598-019-40742-z)
Supplement: Supplementary file 1 — Supplementary Figures [file 41598_2019_40742_MOESM1_ESM.docx]

**Dynamic flood modeling essential to assess the coastal impacts of climate change**

Patrick L. Barnard^1*^, Li H. Erikson^1^, Amy C. Foxgrover^1^, Juliette Finzi Hart^1^, Patrick Limber^1,2^, Andrea C. O’Neill^1^, Maarten van Ormondt^3^, Sean Vitousek^1,4^, Nathan Wood^5^, Maya Hayden^6^ and Jeanne M. Jones^7^

^1^United States Geological Survey, Pacific Coastal and Marine Science Center, Santa Cruz, CA, 95060, USA

^2^Coastal Carolina University, Department of Marine Science, Conway, SC, 29528, USA

^3^Deltares-Delft Hydraulics, Delft, The Netherlands

^4^University of Illinois at Chicago, Department of Civil and Materials Engineering, Chicago, IL, 60607, USA

^5^United States Geological Survey, Western Geographic Science Center, Portland, OR, 97201, USA

^6^Point Blue Conservation Science, Petaluma, CA, 94954, USA

^7^United States Geological Survey, Western Geographic Science Center, Menlo Park, CA, 94025, USA

*Corresponding author, email: pbarnard@usgs.gov

**Supplementary Figures**


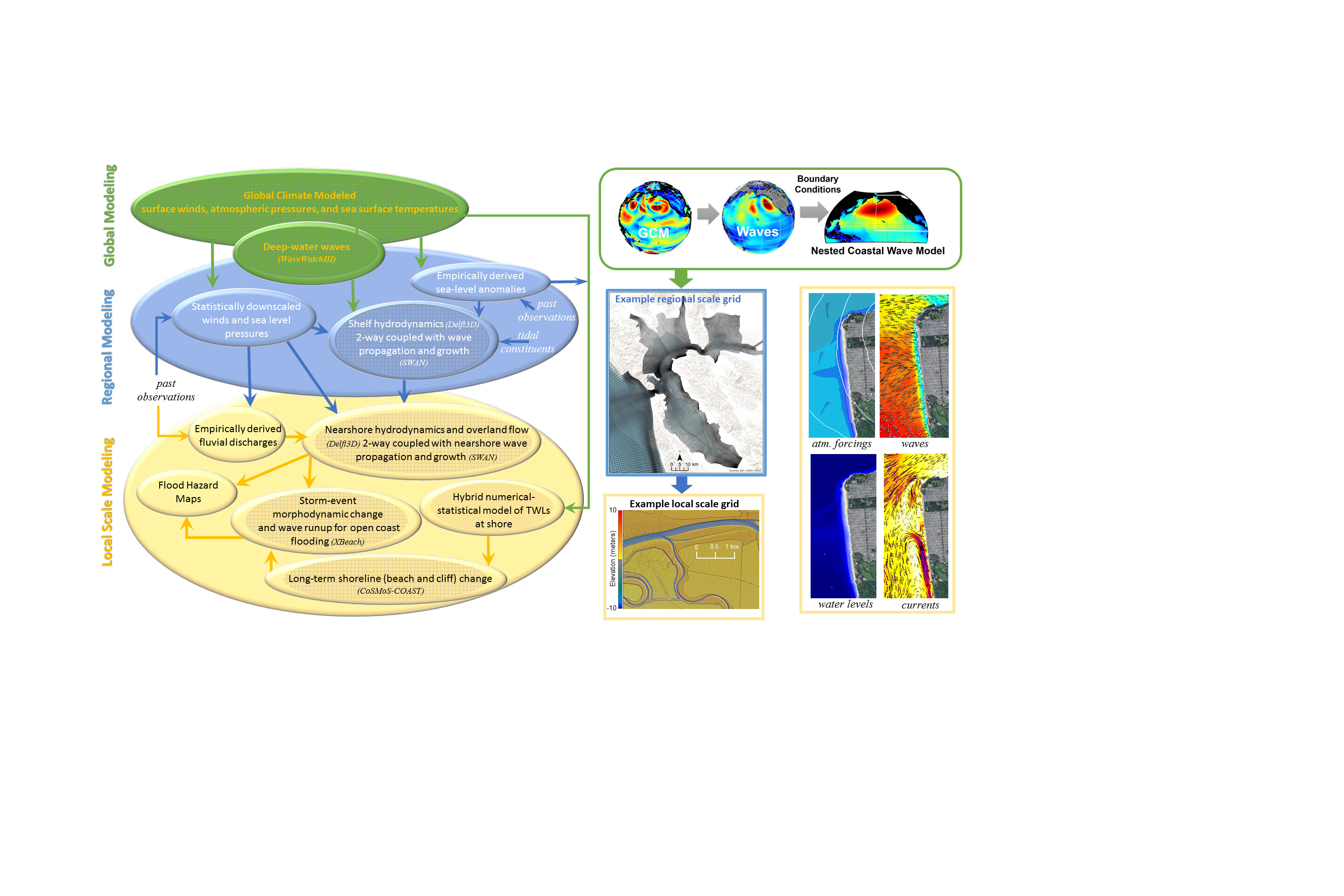


**Figure S1.** **Detailed CoSMoS model framework.** CoSMoS features a series of coupled numerical models that translate the physical forcing derived from Global Climate Models into local coastal flood projections, incorporating sea level rise, tides, seasonal effects, storm surge, fluvial discharge, and waves, as well as short- and long-term coastal change. Hashed ovals denote the use of numerical models. The hybrid numerical-statistical model is used to develop continuous time-series of total water levels at the shore using a linear superposition of wave runup (maximum excursion that waves reach onshore), storm surge, and sea levels, in contrast to the numerically modeled flood maps which simulate non-linear interactions between changing water depths and waves. Software citations: WaveWatch3 – v. 3.14, <polar.ncep.noaa.gov/waves/wavewatch> (upper right); Delft3D and SWAN – Delft3D v. 4.01, <oss.deltares.nl/web/delft3d> with Matlab v. 2015b (<mathworks.com>) and Global Mapper v. 17 (<bluemarblegeo.com>) used to generate images (lower right 3 panels).

**
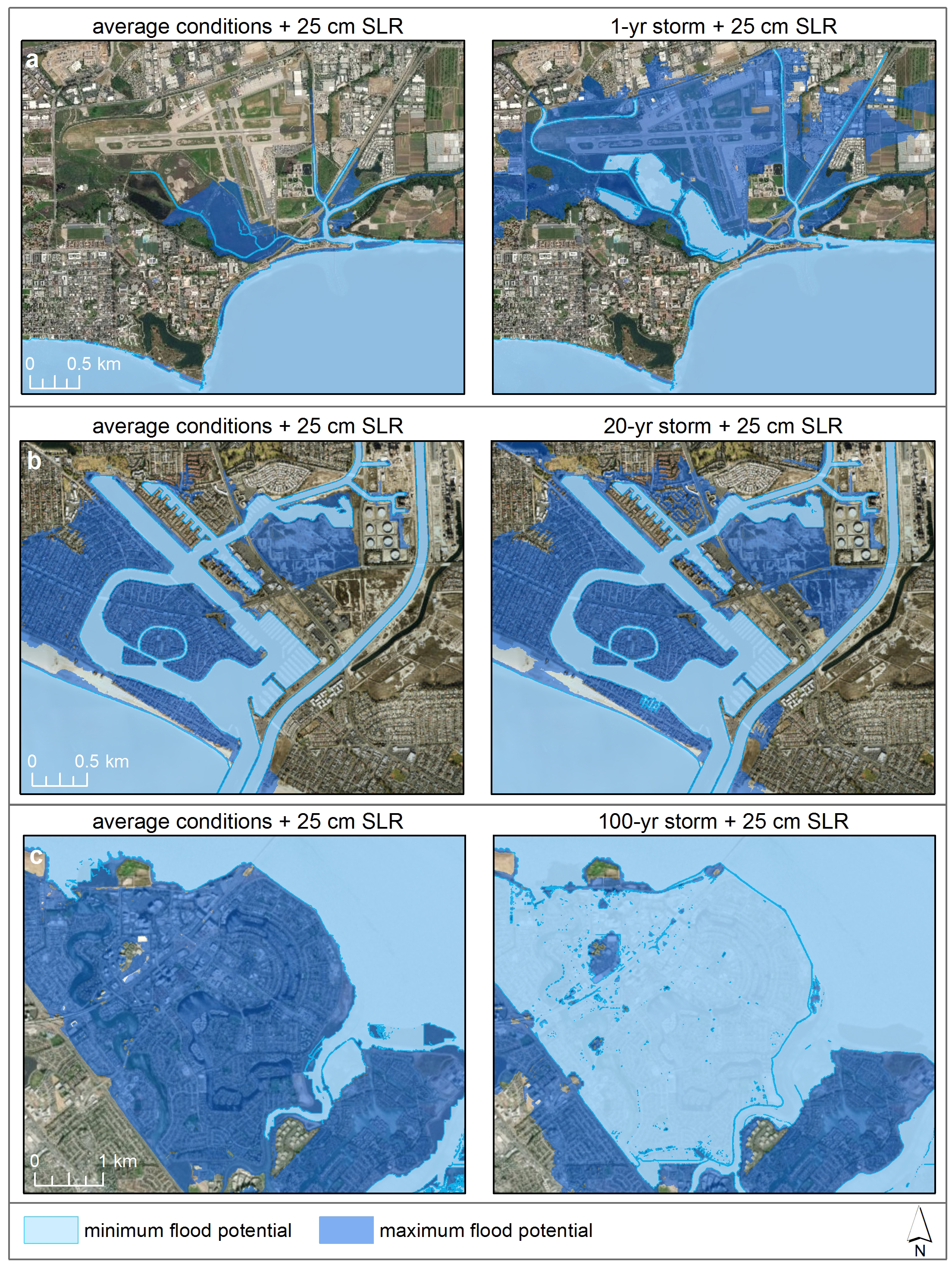
**

**Figure S2. Examples of the coastal flooding uncertainty range for 0.25 m of sea level rise and storms.** These examples, which have the same geographic extent as Figure 4 where median flood projections are depicted, show minimum flood potential and maximum flood potential based on the uncertainty in flood extent due to uncertainty in topographic elevations, water level predictions, and vertical land motion (see Methods for more details). The left hand series of panels depicts projected coastal flood uncertainty range during average conditions (i.e. daily/background conditions with spring tide), and the right side select storm scenarios: (a) Santa Barbara Municipal Airport, (b) Alamitos Bay, Long Beach, and (c) Foster City. (Figure generated using ArcGIS v. 10.4.2, [www.esri.com](http://www.esri.com). Local basemaps from <http://services.arcgisonline.com/arcgis/services>, World_Terrain_Base and ESRI_Imagery_World_2D, accessed 2 Oct 2018.)


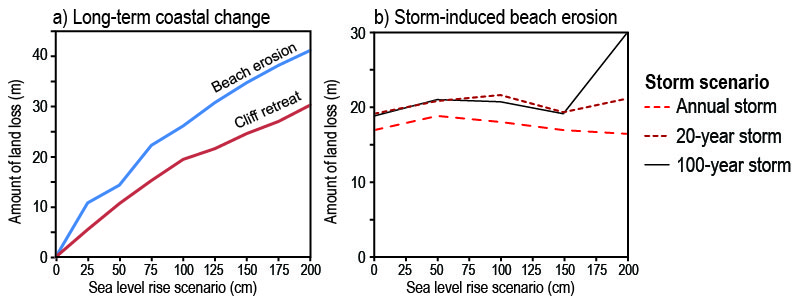


**Figure S3. Coastal change projections.** (a) Long-term coastal change projections for beaches and cliffs, and (b) Storm-induced erosion. Both are integrated into the coastal flooding projections for each of the SLR and storm scenarios modeled by CoSMoS.


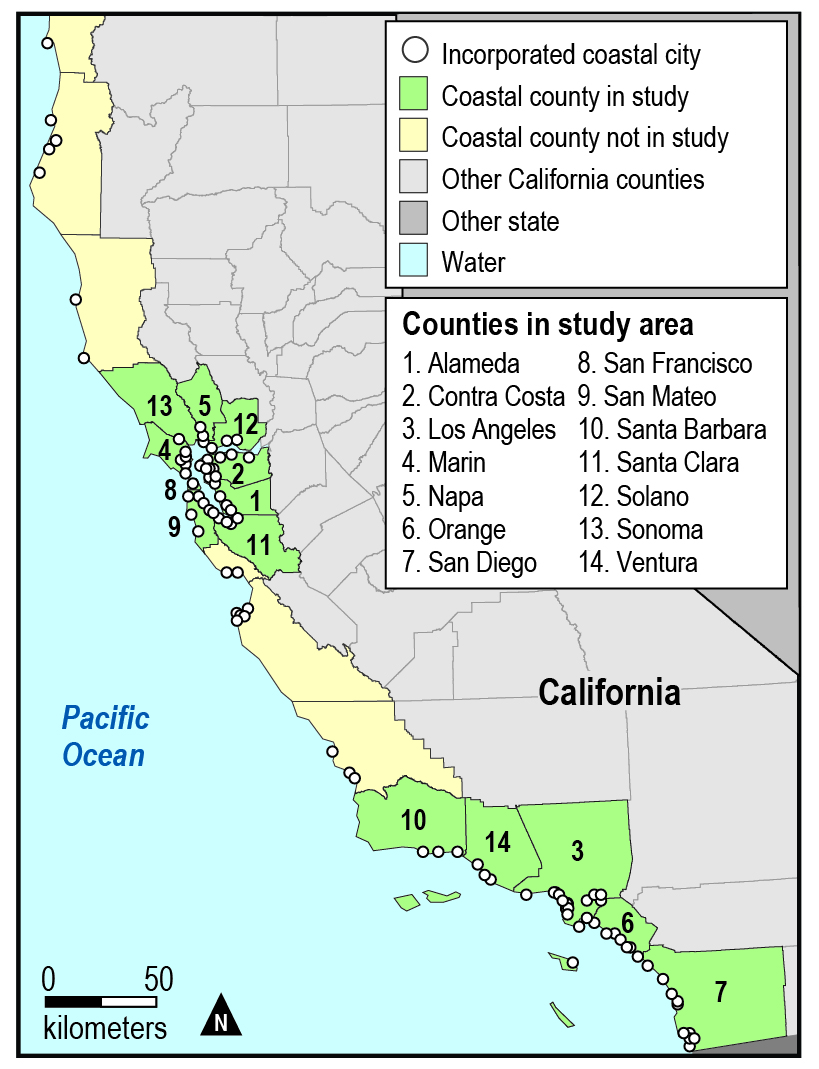


**Figure S4. Study area highlighting the jurisdictions included in the socioeconomic analysis.** Map of California displaying the counties and municipalities that were delineated in the socioeconomic exposure analysis and served up in the Hazard Exposure Reporting and Analytics (HERA) web tool. The counties included in this analysis comprise 95% of the state’s coastal county residents.
